# Deciphering the Role of Ion Channels in Early Defense Signaling against Herbivorous Insects

**DOI:** 10.3390/cells10092219

**Published:** 2021-08-27

**Authors:** Akanksha Gandhi, Rupesh Kariyat, Amaravadhi Harikishore, Marzieh Ayati, Anirban Bhunia, Nirakar Sahoo

**Affiliations:** 1 Department of Biology, University of Texas Rio Grande Valley, Edinburg, TX 78539, USA; akankshagandhi037@gmail.com (A.G.); Rupesh.kariyat@utrgv.edu (R.K.); 2 School of Biological Sciences, Nanyang Technological University, 60 Nanyang Drive, Singapore 637551, Singapore; AMAR0002@e.ntu.edu.sg; 3 Department of Computer Science, University of Texas Rio Grande Valley, Edinburg, TX 78539, USA; marzieh.ayati@utrgv.edu; 4 Department of Biophysics, Bose Institute, Kolkata 700054, India; anirbanbhunia@gmail.com

**Keywords:** reactive oxygen species, herbivory, membrane potential, ion channel

## Abstract

Plants and insect herbivores are in a relentless battle to outwit each other. Plants have evolved various strategies to detect herbivores and mount an effective defense system against them. These defenses include physical and structural barriers such as spines, trichomes, cuticle, or chemical compounds, including secondary metabolites such as phenolics and terpenes. Plants perceive herbivory by both mechanical and chemical means. Mechanical sensing can occur through the perception of insect biting, piercing, or chewing, while chemical signaling occurs through the perception of various herbivore-derived compounds such as oral secretions (OS) or regurgitant, insect excreta (frass), or oviposition fluids. Interestingly, ion channels or transporters are the first responders for the perception of these mechanical and chemical cues. These transmembrane pore proteins can play an important role in plant defense through the induction of early signaling components such as plasma transmembrane potential (V_m_) fluctuation, intracellular calcium (Ca^2+^), and reactive oxygen species (ROS) generation, followed by defense gene expression, and, ultimately, plant defense responses. In recent years, studies on early plant defense signaling in response to herbivory have been gaining momentum with the application of genetically encoded GFP-based sensors for real-time monitoring of early signaling events and genetic tools to manipulate ion channels involved in plant-herbivore interactions. In this review, we provide an update on recent developments and advances on early signaling events in plant-herbivore interactions, with an emphasis on the role of ion channels in early plant defense signaling.

## 1. Introduction

Plants regularly encounter a wide range of abiotic and biotic stresses in nature. Abiotic stress includes drought, salinity, extreme temperatures, radiation, floods, and heavy metals, whereas biotic stressors include insect, animal herbivores, and microbial pathogens. Plant and insect-herbivore interactions are among the most significant species interactions found in nature [1,2], and it is estimated that, annually, herbivory causes a 20% loss in the total productivity of agricultural crops [3]. However, plants are not totally defenseless against herbivory and are able to perceive and respond to this onslaught. They can perceive the insect attack through both mechanical and chemical cues. Mechanical signals are elicited through the damage caused by herbivores by piercing, chewing, or biting of plant tissues, and chemical signals are relayed via herbivore-associated elicitors (HAEs) such as oral secretions (OS) or regurgitant, insect excreta (frass), or oviposition fluids, to name a few [4,5]. Plants not only actively respond to herbivory, but also initiate a series of biochemical responses following the perception of herbivory. These biochemical cascades are initiated through ion channels that control the changes in the plasma membrane potential (V_m_), generation of reactive oxygen species (ROS), cytosolic calcium fluxes, and ultimately induce plant defense genes to mount a multi-layered defense response that can act at both local and systemic levels [4,6,7,8,9,10]. In recent years, there have been several reviews on plant-herbivore interactions [4,5,7,11,12,13,14,15,16,17,18,19,20,21,22,23,24,25,26]. Here we complement these existing reviews with current research and recent discoveries on plant-herbivore interactions, focusing on early plant defense signaling, with a particular emphasis on ion channels involved in early plant defense signaling.

## 2. Long-Distance Communication in Plant Defense

During herbivory, the damaged areas of the plant need to inform the rest of the plant to keep them ready for the imminent herbivory threat. Therefore, plants need to alert their unaffected parts by sending long-distance signals from the site of damage to various parts of the plant to appraise the threat. Plants respond to diverse stimuli by communicating amongst cells from distinct tissues or organs, a process called systemic signaling [27]. Studies have revealed the existence of complex regulatory mechanisms that allow the plant to activate resistance in systemic tissues, commonly referred to as systemic acquired resistance (SAR) [28]. SAR is characterized by a more potent and faster response to future encounters with microbes, insects, or abiotic stress.

Considerable progress has been made in understanding this intricate relationship between plants and herbivores with a plethora of field and lab studies. These include studies that have dissected pairwise interactions between a specific herbivore and its host; interactions at species, genus, and community levels with multiple hosts and herbivores; and studies examining plant defense signaling networks through molecular genetics genomics, to name a few [29,30]. However, our knowledge of how plants perceive these cues and how that leads to specific and tightly regulated defense responses is still in its infancy. It has been proposed that following the insect attack, the foremost event is the recognition of the cue and its perception by specific membrane receptors and the transduction of these signals into the plant cell. These cues are termed as “early defense signaling molecules” such as the depolarization of plasma membrane along with the generation of secondary messengers such as cytosolic Ca^2+^ [31], reactive oxygen species (ROS), and reactive nitrogen species (RNS) [32,33,34,35] that contribute to plant defense signal transduction events.

**Figure 1:**
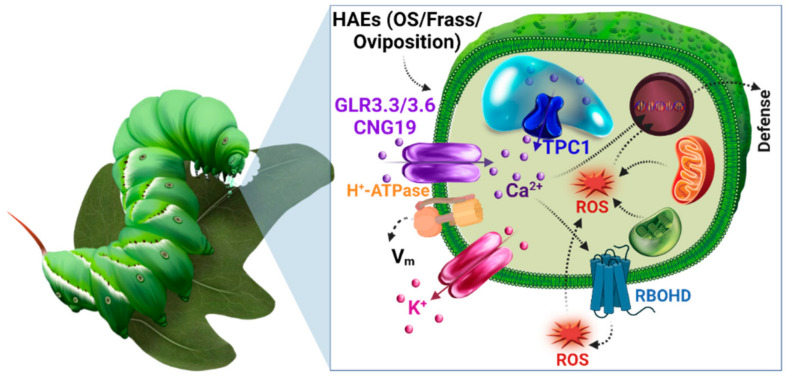
Initiation of early defense signaling mechanisms in response to insect herbivore attack. Schematic diagram showing herbivore *M. sexta* feeding induced signaling events, which include the perception of HAEs such as OS, frass, and oviposition by specialized receptors on the outer plasma membrane, which trigger modulation of V_m_ via H^+^-ATPase and Ca^2+^ ion influx into the cell via Ca^2+^ channels, GLR3.3/3.6 and/or CNGC19. The increase in cytosolic Ca^2+^ may trigger the further release of vacuolar Ca^2+^ via the TPC1 channel. The subsequent release of Ca^2+^ may activate nicotinamide adenine dinucleotide phosphate (NADPH oxidase) and respiratory burst oxidase homologues (RBOHDs), leading to ROS generation, and induction of plant defense responses. Illustration by Annette Diaz.

Long-distance communication in plants has been linked with ion channels or membrane transporters. These are transmembrane pore proteins involved in the movement of ions across the cell membrane. In recent years, with electrophysiological tools, the research on ion channels in plants has been gaining momentum. Studies have reported that ion channels facilitate long-distance communication via V_m_, Ca^2+^, and ROS (Figure 1). Ion channels have been shown to mediate systemic signaling by modulating the influx of ions into different plant tissues [36]. They sense signals from the functional cells at the site of herbivory to activate other cells, which in turn relay this signal to induce defense responses. For example, a recent study [37] identified glutamate receptor-like channels (GLRs) in *Arabidopsis thaliana* that are related to mammalian ionotropic glutamate receptors, play a role in Ca^2+^ signaling during herbivory, nutrient transport, root gravitropism, and plant defense [38,39]. However, in mammals, these channels are involved in neurotransmission, and their openings are stimulated by glutamate binding to the postsynaptic neuron, resulting in Ca^2+^ and other cations influx. The signal is transmitted because of voltage changes caused by ion flux [40]. Remarkably, these GLRs are also responsible for long-distance Ca^2+^ transmission in plants in response to herbivory or mechanical injury, efficiently communicating herbivore attacks to surrounding cells.

There has been considerable research on identifying the factors that are involved in long-distance signaling. Plants can appraise their unaffected parts by extensive network of intracellular regulators, V_m_, Ca^2+^, and ROS [18,41]. The transmission rate of all these waves ranges from ~100 to >1000 µm/sec [41,42]. The process starts with the propagation of long-distance electrical signals as a result of variation in membrane potential due to potassium (K^+^) and Ca^2+^ flux. Variation in V_m_ is critical for plant wounding responses [43]. Finally, Ca^2+^ and ROS, versatile secondary messenger, were generated that plants use to sense and transform environmental stimuli into an adaptive intracellular response [44]. Insect feeding and OS can lead to changes to the cytosolic Ca^2+^ concentration, and these spatiotemporal variations have been shown to yield Ca^2+^ signatures [45,46,47,48]. On the other hand, ROS are extremely reactive and hazardous chemicals formed from oxygen. Among them are O_2_, H_2_O_2_, and OH^−^. ROS which has been demonstrated to act as a self-propagating long-distance and fast wound signal [49]. Throughout this review, we will focus on the role of ion channels, V_m_, Ca^2+^, and ROS in plant response to herbivory and provide an overview of what is currently known about the role of ion channels in plant-herbivore interactions.

## 3. Membrane Potential (V_m_)

The V_m_ is an electrical potential of the cell membrane that is maintained via the balance of ion fluxes across the plasma membrane. V_m_ indicates whether a cell is excited or not. It is responsible for generating action potentials in tissues, muscles, and nerves in animals and plays a crucial role in diverse biological functions such as biological sensing, hearing, cell cycle, proliferation, contractility, and circadian rhythm, to name a few [50]. Unlike animals, plants use V_m_ to regulate plant cellular functions such as maintaining turgor pressure, osmotic balance, and stomatal closure. There is no net flux of ions through the membrane when in equilibrium, called the resting membrane potential. Changes in the resting membrane potential will occur due to an unbalanced movement of ions, thus leading to V_m_ being more positive (depolarization) or more negative (hyperpolarization). In general, plants maintain a negative resting membrane potential in the order of −110 to −150 mV [51,52]. It has been reported that the signal transduction mechanism of plants to respond to minor changes in V_m_ leads to plant defense responses. The way plants sense insect cues and initiate defense responses has been a point of interest for many years. One hypothesis that has evolved by studying cellular responses following herbivory suggests that the first event following herbivory generates the fluctuation in V_m_ [53]. Maffei et al. [43] has also demonstrated that both mechanical wounding and OS of cotton leafworm (*Spodoptera littoralis*) alter V_m_ in lima bean (*Phaseolus lunatus* L.) at increasing distances of 5, 30, and 60 mm from the bite zone. V_m_ depolarization was observed within the first 15 min of feeding by *S. littoralis* in the palisade cells. The effect of *S. littoralis* regurgitant and its components were also tested on V_m_ in *P. lunatus* leaf and the results showed that V_m_ alterations were independent of regurgitate concentration. In addition, they also examined changes in V_m_ in response to the application of various H_2_O_2_ concentrations to mechanically damaged and herbivore-wounded *P. lunatus* leaves. H_2_O_2_ treatment induced a robust V_m_ that was significantly greater in herbivory-wounded plants than in mechanically injured leaves [54].

Bricchi et al. [55] studied V_m_ alterations in wild-type and plasmodesmata mutated *A. thaliana pdko3* lines; plasmodesmata are channels within the plant cell that allow chemicals to pass through, establishing a pathway for cell-to-cell communication. A strong V_m_ depolarization occurred in wild-type *A. thaliana* plants within 7 to 8 min after herbivory, but the *pdko3* mutant did not exhibit V_m_ depolarization in response to herbivory or application of OS from *S. littoralis*. However, Ca^2+^ elevation was observed in both wild types as well as in *pdko3* mutant. This observation ruled out the possibility of Ca^2+^ channels being involved in V_m_ depolarization. To dissect the dependence of V_m_ depolarization on potassium (K^+^) channels, the K^+^ channel activity was measured using fluorescent indicator FluxOR^TM^. A significant increase in K^+^ channel activity was observed in wild-type plants, whereas a complete loss of K^+^ channel activity was observed in *pdko3* plants. This finding also suggests that K^+^ channels are involved in V_m_ depolarization and supports the hypothesis that plant cells respond to OS by a V_m_-mediated signal transduction pathway.

The fluctuation in V_m_ has been known to be induced by the binding of specific components from herbivore OS with the receptors present at the plasma membrane [56]. These components can alter ion channel activities, causing an imbalance in ion movement, which influences the membrane potential of the plasma membrane [43]. A study by Mohanta et al. [57] showed that Kew tree (*Ginkgo biloba*), a living fossil plant, responds to *S. littoralis* herbivory by inducing V_m_ depolarization, which was evident up to 6 h. Another study using *A. thaliana* also showed that the extent of V_m_ depolarization was the same for *S. littoralis*, green peach aphid (*Myzus persicae)*, and the plant pathogenic bacteria *Pseudomonas syringe*, but the timing of the occurrence of V_m_ depolarization was different for each of these biotrophs. Moreover, the magnitude of early defense response depends upon the amount of tissue damage by the biotroph. V_m_ depolarization was rapid upon the attack of chewing herbivore, *S. littoralis* (30 min to 2 h), as it caused substantial tissue loss, since it consumed large amounts of leaf tissue. On the other hand, less damage was observed by a phloem feeder, *M. persicae* (4 to 6 h), that delayed the plant defense response since phloem feeders with sucking mouthparts feed on vascular tissues without visible tissue damage as observed with chewing herbivores [58]. It is apparent that Ca^2+^ and ROS generation are directly tied to V_m_ when herbivores interact with plants, and V_m_ is essential for plant defense responses.

## 4. Calcium (Ca^2+^)

Ca^2+^ is a ubiquitous signaling molecule in plants. It functions as a secondary messenger in cellular pathways that regulate plant growth and development, cell polarity, cytoskeleton organization, ion transport across membranes, stomatal regulation, root growth, fertilization, nutrient signaling, and plant immunity [59]. Consequently, each of these processes has its own “Ca^2+^ signature,” linked with distinct fluctuations in Ca^2+^ concentration in the cytosol and sometimes in a particular intracellular compartment. Therefore, Ca^2+^ fluxes, especially oscillations between calcium stores and the cytosol, are important for cell signaling [60,61,62].

In plants, the cytosolic Ca^2+^ concentration is maintained at or below 100 nM; however, the majority of Ca^2+^ is stored in the apoplast, vacuole, endoplasmic reticulum (ER), and Golgi apparatus. The apoplast serves as the first Ca^2+^ reservoir of a cell that can store 0.33 mM free resting Ca^2+^ and the first area that responds to stimuli, while the vacuole serves as the largest Ca^2+^ pool of a cell that can store up to 0.2–5 mM free resting Ca^2+^ [60,63].

The Ca^2+^ signature plays an important role in long-distance signal transduction during herbivore attack through which HAEs such as OS, oviposition, and frass is sensed by the cell membrane, and then, a Ca^2+^ is rapidly propagated in the cytosol and travels throughout the plant to induce defense responses. The shaping of this “Ca^2+^ signature” during plant-herbivore interactions is achieved through the amplification and integration of Ca^2+^ signals. The amplification step is mediated via specific ion channels or transporter proteins and enhances Ca^2+^ fluxes at sites of herbivore attack, whereas the integration step is mediated via Ca^2+^ sensor proteins, which allow efficient transmission of Ca^2+^ signals from one cell to another in a tissue or organ. Herbivory induces Ca^2+^ entry from the apoplast to the cytosol via plasma membrane Ca^2+^ channels which stimulates Ca^2+^ signals in the cytosol leading to the amplification of Ca^2+^ signals. The localized Ca^2+^ signals from the cytosol are distributed throughout the whole plant. In this way, amplification, and integration of Ca^2+^ signals constitute two important ways by which “Ca^2+^ signature” contributes as a signaling molecule during plant-herbivore interactions [64].

The amplification of intracellular Ca^2+^ signal requires selective Ca^2+^ sensor proteins that respond to changes in cytosolic Ca^2+^ levels and encipher the frequency, amplitude, and signal localization of Ca^2+^ signatures. It is estimated that *A. thaliana* contains around 250 Ca^2+^ sensor proteins [65]. These can be classified into three main categories: (1) the calcineurin B-like proteins (CBLs) [66]; (2) the calmodulin (CaM), and calmodulin-like proteins (CMLs) [67]; and (3) the Ca^2+^ dependent protein kinases (CPKs) and the Ca^2+^ and calmodulin-dependent protein kinase (CCPK) [68]. All of these sensors contain EF-hand motifs, which enable Ca^2+^ binding and cause conformational changes in their structure [69].

CaM functions as a sensor relay protein since it lacks an enzymatic function. The *Arabidopsis* genome has seven calmodulin genes encoding four different isoforms (CaM1/4; CaM2/3/5; CaM6; and CaM7) [70]. CaM/CaM-like proteins (CML) regulate a variety of transcription factors, protein kinases, phosphatases, metabolic enzymes, ion pumps, and ion exchangers [71]. *A. thaliana* signal responsive (AtSR1) proteins [67], also known as CaM-binding transcription activators (AtCAMTAs) [72], have been shown to participate in wound-mediated defense responses. *Atsr1* mutants of *A. thaliana* were sensitive to attack by dark winged fungus gnats (*Bradysia impatiens*), suggesting the role of CaM as an important sensor in the early stages of the insect-plant attack [73]. Along with CaM, the plant has CML that undergo secondary structural changes in response to Ca^2+^ binding and act as Ca^2+^ relays/sensors [74]. CML and CAM share a 16% amino acid sequence similarity and include two to six EF-hand motif [70]. CML42 gene expression was shown to be increased in *A. thaliana* upon *S. littoralis* OS treatment, implying a function in early defense plant signaling [75]. CPKs have been classified as sensor responders because they combine a Ca^2+^ binding domain and a serine/threonine kinase domain into a single protein that performs the fundamental function of converting Ca^2+^ signals to phosphorylation events [76,77]. *A. thaliana* contains 34 CPK family genes that play a role in plant defense responses. CPK 3 and CPK 13 both participate in signaling after Ca^2+^ influx upon *S. littoralis* attack through regulation of plant defensin gene (PDF1.2) by phosphorylation of the transcription factor, HsfB2a [78]. The *cpk3* and *cpk13* mutants had much lower transcript levels of the plant defensin gene PDF1.2 in comparison to wild-type plants.

### Tools Used to Monitor Ca^2+^ Signaling in Plant-Herbivore Interactions

In recent years, the research on Ca^2+^ signaling has gained momentum with the advance in Ca^2+^ imaging techniques. Therefore, it is important to discuss different plant Ca^2+^ imaging methods, which are widely used in the context of plant-herbivore interactions to observe and record cytosolic Ca^2+^ concentration in herbivore-infested plants. These techniques include the use of Ca^2+^ sensing fluorescent dyes and genetically encoded Ca^2+^ indicators. Various fluorescent Ca^2+^ sensing dyes, such as Fluo-3, Calcium Orange, etc., have been used to investigate the dynamics of cytosolic Ca^2+^ signals in plant- herbivore interaction [33,43,55,57,58,79,80,81]. For example, the Ca^2+^ indicator Ca^2+^ orange was utilized to identify changes in cytosolic Ca^2+^ concentrations in *P. lunatus* following *S. littoralis* herbivory. The changes in Ca^2+^ concentration were compared in response to a single wounding (MD) event, continual mechanical damage caused by a robotic worm (MecWorm, MW), and herbivory. After 30 min, a considerable increase in Ca^2+^ fluorescence was observed due to herbivory in the wounding zone, which persisted for 4 h, but in MD and MW plants, just a faint fluorescence was noticed [33]. Even though these dye-based markers have been demonstrated to be quite effective, these Ca^2+^ sensing dyes have some limitations, including toxicity, fragility, low fluorescence signals, and they cannot be imaged in living plants without permeabilization. To overcome these limitations, researchers have initiated research on the use of genetically encoded Ca^2+^ indicators. The most widely used Ca^2+^ imaging method includes genetically encoded Ca^2+^ indicators, such as GCaMP, Yellow Cameleon (YC) Ca^2+^-sensors. The Ca^2+^ sensors were developed from GFP by combining them with calmodulin. These Ca^2+^ sensors can be expressed in the whole plant and are functional throughout the entire plant. Therefore, it can be used to monitor cytosolic Ca^2+^ in plants subjected to various herbivore attack conditions [37,42,82,83,84]. For example, Toyota et al. [37] showed that the *P. rapae* caterpillars induced cytosolic Ca^2+^ responses in the leaves of *A. thaliana* can be monitored with GCaMP3. This study reported that the increases in cytosolic Ca^2+^ concentration were associated with ion influx through plasma membrane Ca^2+^ channels such as GLR3.3/GLR3.6. Another example is Verrillo et al. [83], who showed that Ca^2+^ induction could be monitored with YC3.60, a YC-based Ca^2+^ sensor, following application of *S. littoralis* OS on mechanically damaged *A. thaliana* leaves. By using these tools, it is now possible to study the dynamics of Ca^2+^ signaling in plant-herbivore interactions at single-leaf, whole-plant, and whole-plant-insect herbivore attack conditions.

Intracellular Ca^2+^ level is controlled by the influx of Ca^2+^ ions from extracellular through apoplastic and vacuolar membranes. Therefore, plant ion channels play an important role in regulating plant development and the perception of many stimuli, including herbivory.

## 5. Plant Ion Channels

**Figure 2:**
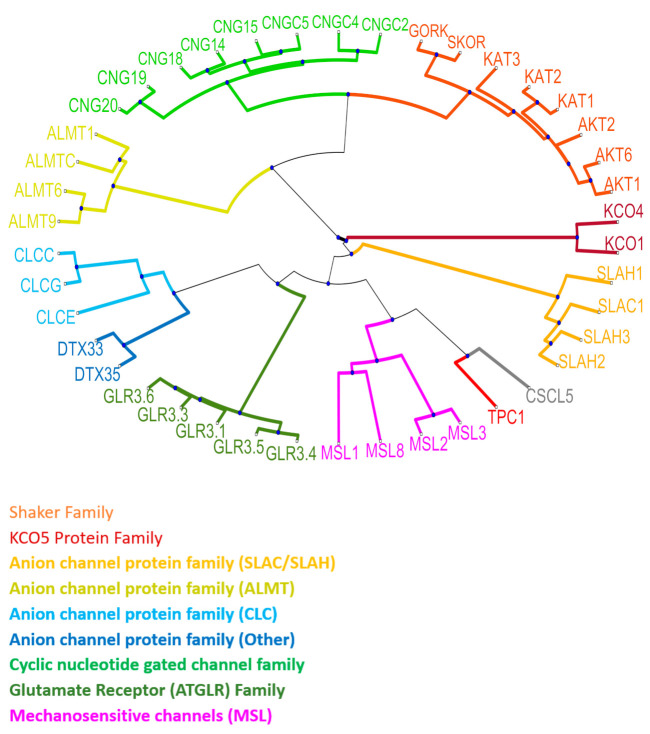
Phylogeny of plant ion channels. Representation of the phylogenetic tree of plant ion channels listed in Pantoja, 2020 [88], based on the analysis of protein homologs extracted from Uniprot.org. Progressive alignment and BLOSUM30 scoring method were used for multiple sequence alignment. The distance between the aligned sequences was calculated using Jukes-Cantor method. The phylogenetic tree was created by using the distance matrix. Unweighted pair group method average (UPGMA) was used to calculate group distance in the tree. Different colors represent different families of ion channels.

Ion channels are macromolecular pores in the membrane that regulate the influx and efflux of ions across the membrane at a rate of 10^6^ ions per second. Ion channels can control ion fluxes in their target compartment and, thus, modify cellular homeostasis, and are vital in osmoregulation, development, signaling, mobility, and uptake of nutrients by the root and long-distance communication [85,86]. The first plant ion channel discovered, in 1984, is a K^+^ channel, Stelar K^+^ outward rectifier (SKOR) [87]. The last two to three decades have seen a dramatic increase in the number of ion channel subfamilies and their diverse functions. A large proportion of plant ion channel families have an analogous expression in animals. Ion channels are arranged into large families and are generally classified as cation, anion, or ligand-gated channels. Cation channels include voltage-gated K^+^ channels such as the shaker family (AKT1, AKT2, AKT6, KAT1, KAT2, KAT3, GORK, and SKOR; K^+^ transport), tandem pore, and two-pore K^+^ channels (TPK1^,^ TPK4; K^+^ transport and TPC1; Ca^2+^ and other cation transport), are responsible for permeation of K^+^ ion across the plasma membrane and tonoplast membrane. Anion channels include slowly activating anion channels (SLAC1, SLAH1, SLAH2, SLAH3; Cl^−^/NO_3_^−^ transport), aluminum-activated malate transporters (ALMT1, ALMT6, ALMT9, ALMT12; Malate, Cl^−^ transport), chloride channels/transporters (CLCc, CLCg, CLCe; Cl^−^ transport), and detoxification efflux carrier (DTX33, DTX35; Cl^−^ transport). Ligand-gated channels include cyclic nucleotide-gated channel (CNGC2, CNGC4, CNGC5, CNGC14, CNGC15, CNGC18, CNGC19, CNGC20; Ca^2+^/Ba^2+^ transport) and glutamate receptor-like channels (GLR3.1, GLR3.3, GLR3.4, GLR3.5, GLR3.6; Ca^2+^ and other cations transport) [88] (Figure 2). These channels are responsible for setting up membrane potential, signal transduction, water, and solute transport [89], stomatal opening and closure [90,91], pollination [92], salt tolerance [93], and plant defense [94], to name a few. However, four distinct families of Ca^2+^-transporting ion channels have been shown to play a role in plant-herbivore interactions, including cyclic nucleotide-gated channels (CNGC19) [95,96], glutamate receptor-like channels (GLR3.3, GLR3.6) [37,42,97], two-pore channel 1 (TPC1) [59,84,98], and annexins (ANNEXIN 1) [99,100].

### 5.1. Cyclic Nucleotide Gated Channels (CNGC)

The cyclic nucleotide-gated channels (CNGCs) are ligand-gated Ca^2+^ channels, first discovered in retinal photoreceptors and olfactory neurons [101]. They play a role in signal transduction in animals and are also present in other non-neuronal tissues [102]. These ion transport proteins have also been identified in plants [74,103,104] and have been known to be involved in a variety of biological processes, ranging from plant development and stress tolerance, disease resistance [105,106], thermal tolerance [107], and salt stress [108]. These channels are typically localized at the plasma membrane and in the model plant *A. thaliana,* which consists of 20 family members [109].

**Figure 3:**
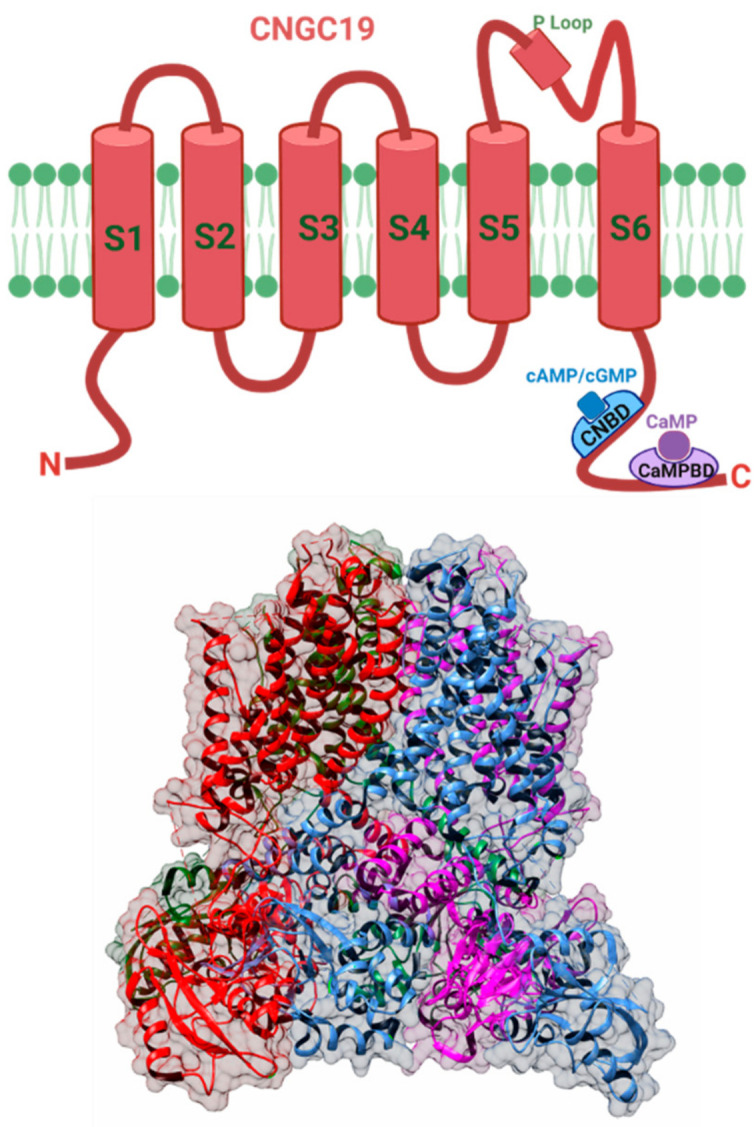
Putative structure of CNGC19 channel. (Top) Schematic cartoon representation of CNGC19 channel subunit showing six membrane-spanning regions (S1–S6) and a large pore domain (S5–S6). Functionally relevant sites in the *C*-terminus consist of a CNB, cyclic nucleotide-binding domain which can bind cAMP/cGMP, and a CaMBD, calmodulin-binding domain which can bind calmodulin. The functional channel is formed by four subunits. (Bottom) The structure of CNGC19 has not been solved to date but is likely to show similarities with the animal CNG family of channels. Therefore, the structure shown in the figure is an approximation based on homology to other channels. The predicted CNGC19 secondary 3D structure model, showing four subunits in transparent surface view, was developed from the closest homolog PDB structure, 5VA1 (human ether-a-go-go related K^+^ channel) using PHYRE 2.0 program. The image was prepared using Chimera software [122]. Created with BioRender.com (accessed on 30 August 2021).

CNGC channel is composed of four subunits, and each of these subunits consists of six membrane-spanning regions and a pore domain [110]. There is a cyclic-nucleotide binding (CNB) and a calmodulin-binding domain (CaMB) present at the *C*-termini of the channel (Figure 3) [111]. In contrast, the animal system has a CaMB domain at the *N*-termini [112,113]. The plant and the animal CNGC differ in their pore amino acid sequence as well as the selectivity for various cations [105,114]. The amino acids that form the CaM binding domain overlap with the polypeptide region that includes the CNBD [115]. This overlapping affects the channel activation as the binding of CaM at the C termini hinders cyclic nucleotide-binding, suggesting variability in plant and animal CNGC channel regulation [116,117]. These channels are activated by the binding of cyclic nucleotides such as cAMP (cyclic adenosine monophosphate) and cGMP (cyclic guanosine monophosphate) [118,119,120], and inhibited by calmodulin binding [121]. These channels also show similarity with shaker-like K^+^ channels [105]. Patch-clamp recordings on plant cell protoplasts membrane directly show that CNGC activation can be achieved by the application of hyperpolarizing potentials (more negative than −120 MV), which allow Ca^2+^ entry into the cell [111,121].

It has been demonstrated that CNGC channels are important in modulating biotic stress responses such as Ca^2+^ influx in plant responses mediated by insect herbivore feeding [95]. A recent study by Meena et al. [96] has shown that the *A. thaliana* CNGC19 is responsible for generating and transmitting Ca^2+^ signals in local and systemic leaves mediated by the herbivore *S. litura*. A loss-of-function CNGC19 mutant in which the Ca^2+^ signals were attenuated was found to be more susceptible to attack by *S. litura*. In addition, jasmonic acid, a key signaling molecule in plant defense, was also observed in lower amounts in the CNGC19 mutant. These results suggest that CNGCs are involved in modulating plant resistance to insect herbivores, thus playing a role in the modulation of plant-herbivore interactions.

### 5.2. Glutamate Receptor-Like Channels

**Figure 4:**
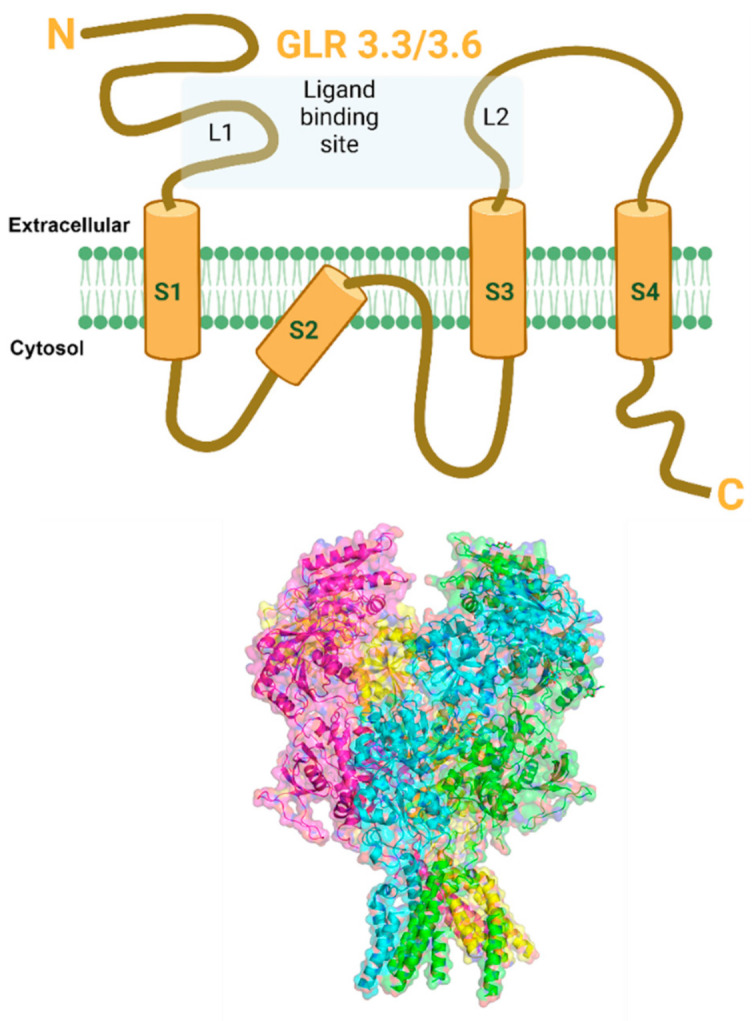
Putative structure of GLR3.3/3.6 channel. (Top) Schematic cartoon representation of GLR3.3/3.6 channel subunit showing extracellular *N*-terminus, four membrane-spanning regions (S1–S4), 2 extracellular ligand-binding sites (L1, L2), and intracellular *C*-terminus. (Bottom) The structure of GLR3.3/3.6 has not been solved to date but is likely to show similarities with the animal NMDA receptor family of channels. Therefore, the structure shown in the figure is an approximation based on homology to other channels. The predicted GLR3.3/3.6 secondary 3D structure model showing four subunits in transparent surface view was developed from closest homolog PDB structure 4TLL (*Xenopus laevis* GluN1/GluN2B NMDA receptor), using PHYRE 2.0 program. The image was prepared using PyMol software (PyMOL Molecular Graphics System, Version 2.4, Schrödinger, LLC, New York, NY, USA). Created with BioRender.com (accessed on 30 August 2021).

Glutamate receptor-like (GLR) is a non-selective ion channel responsible for permeating Ca^2+^ ions across the plasma membrane of animals and plants. Plant glutamate receptor-like (GLR) channels are ionotropic glutamate receptor homologs in mammals (iGluRs). The iGluRs have been extensively studied for their central nervous system and have been known to play a vital role in synaptic transmission, learning, and memory [123,124]. It is intriguing that GLRs also exist in plants despite the absence of the central nervous system [125]. In plants, GLRs play a crucial role in carbon and nitrogen metabolism [126], gravitropism [127], pollen tube growth [128,129], immune defense reactions [38,130,131,132,133], and wound-induced intracellular signaling [97]. *Arabidopsis* consists of 20 GLR genes; each subunit hosts a *N*-terminal domain, two extracellular ligand-binding sites (L1, L2), and transmembrane domains (S1–S4), including a pore region (P) and the *C*-terminal domain [134] (Figure 4). In mammals, iGluRs are divided into three groups according to their sequence diversity and ligand specificities [124]. These include N-methyl-d-aspartate (NMDA), α-amino-3-hydroxy-5-methyl-4-isoxazole propionic acid (AMPA), and Kainate receptors. Plant GluRs share a high degree of similarity with the NMDA receptors that range from 16 to 63% in the ligand-binding domains and the transmembrane domains [135]. These channels are not only present at the plasma membrane but can also be found in chloroplasts, mitochondria [136], and vacuolar membranes [129]. Unlike their mammalian counterparts, the plant GLRs have much broader ligand selectivity. The major difference in plant and animal iGLR is the pore region. These non-selective cation channels are activated by amino acid glutamate, which acts as a metabolite, energy source, and neurotransmitter in animals [137,138].

Electrophysiological studies have shown the involvement of GLRs in inducing a Ca^2+^ influx in plants that leads to the modulation of plant defense signaling to insect herbivores [139,140]. A study by Vasta et al. [140] showed that the application of GLR agonists such as glutamate induced a strong and rapid cytosolic Ca^2+^ increase in tobacco (*Nicotiana tabacum)* var *xanthi* while the application of lanthanum and Ca^2+^ chelator, BAPTA, inhibited glutamate-induced Ca^2+^ increase. This observation suggests that the plant GLR has a role in the modulation of Ca^2+^ influx that ensures plant defense responses against insect herbivores.

GLR3.3 has been implicated in the transmission of signals in the form of Ca^2+^ waves from wounded to unwounded sections of the plant. When *S. littoralis* larvae were allowed to feed on *A. thaliana* wild-type plants, wound-induced surface potential alterations were detected. However, wounding reduced the surface potential alterations in the four GLR mutants GLR3.1, GLR3.2, GLR3.3, and GLR 3.6. [97]. This suggests that GLR3.3 plays an important role in the modulation of plant defense signaling to insect herbivores. Recently, Toyota et al. [37] showed that GLRs are activated by wounding and upon herbivory by cabbage butterfly (*Pieris rapae*) caterpillars in *A. thaliana* leaf expressing genetically encoded Ca^2+^ sensor GCaMP3. The cytosolic Ca^2+^ elevation and subsequent defense gene expression were observed after the application of glutamate and not with other amino acids such as sorbitol. Furthermore, the Ca^2+^ signals were completely abolished in the GLR3.3/GLR3.6 double mutant in *A. thaliana,* suggesting that GLR3.3 and GLR3.6 are essential for transmitting Ca^2+^ signals induced by wounding and herbivory. Another recent study by Shao et al. [42] demonstrated that wounding of the main root at a distance of 2 cm from the root-shoot junction increased the Ca^2+^ elevation and surface wave potential (SWP) in *A. thaliana* expressing calcium sensor GCaMP6. Additionally, the application of glutamate to the wound site in the root induced an increase in Ca^2+^ and SWP in all leaves. Interestingly, in the GLR3.3/GLR3.6 double mutant, this wound and glutamate-induced rise in root to shoot Ca^2+^ was attenuated. This finding suggests that GLR3.3 and GLR3.6 are involved in propagating systemic Ca^2+^ signaling from leaf to leaf and root to shoot. These results provide evidence for the role of plant GLRs in the modulation of Ca^2+^ signaling during plant defense responses against insect herbivores.

### 5.3. ANNEXIN1

**Figure 5:**
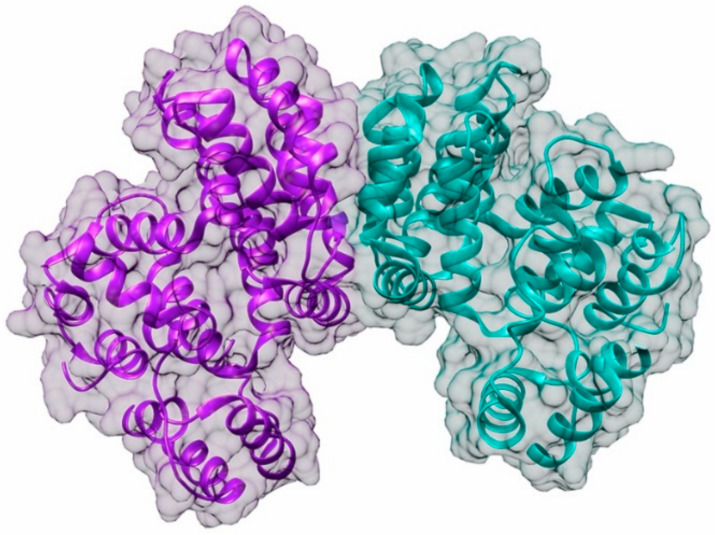
Putative structure of ANNEXIN1 channel. ANNEXIN1 secondary 3D structure model showing two subunits (homodimer) in transparent surface view was developed from PDB structure 1YCN (*Arabidopsis thaliana* ANNEXIN). The presence of Ca^2+^ or H_2_O_2_ appears to be required for homodimerization. The image was prepared using Chimera software [122].

Annexins are the phospholipid-binding proteins and are considered novel mechanosensitive Ca^2+^ channels [141,142]. In animal cells, annexins are present in the cytoplasm and cellular membranes [143]. They are involved in vital cellular processes such as membrane trafficking, ion flux, mitotic signaling, and cytoskeleton rearrangement [143,144]. Eight annexin genes have been identified in *A. thaliana* by genome sequencing [145]. Plant annexins are structurally different from their animal homologs but have a conserved primary sequence. These 32–42 kDa proteins have two major domains: a *N*-terminal head and a *C*-terminal annexin core [143] (Figure 5). The annexin core is composed of four annexin domains (I–IV), each of which is 70 amino acids in length and contains five short helices and a conserved endonexin fold (G-X-G-T-{38-40}-D/E). Ca^2+^ binding activity occurs in type II and III binding sites of annexin proteins [141,143]. Plant annexins have a shorter *N*-terminal region than their animal counterparts [146] and are crucial for actin binding, inhibition of callose synthase, and oxidative stress responses [147,148,149,150]. The functional diversity of annexins is due to the variable *N*-terminal region that interacts with other proteins.

A recent study by Malabarba et al. [100] reported the role of ANNEXIN1 (ANN-1) in initiating systemic defense in *A. thaliana* in response to Egyptian cotton leafworm (*S. littoralis*) herbivory. The study found that annexin 1 was responsible for inducing cytosolic free Ca^2+^ elevation upon wounding and simulated herbivory in *A. thaliana*. ANN-1 knock-out and ANN-1 overexpressing lines were employed in this work to evaluate their role in herbivory-mediated Ca^2+^ signaling. The result showed that in the ANN-1 deletion line, the increase in cytosolic Ca^2+^ upon herbivory by *S. littoralis* was impaired, and the larvae gained more weight than those fed on wild-type plants. On the other hand, weight increase was significantly lower in larvae that fed on the ANN-1 overexpressed line compared to the wild type. Additionally, jasmonate accumulation and defense responses were diminished in ANN-1 systemic leaves, demonstrating that ANN-1 is involved in systemic cytosolic Ca^2+^-dependent jasmonate induction. This finding suggests that ANN-1 modulates plant defenses against herbivore damage through the Ca^2+^-dependent jasmonate signaling pathway and is required for systemic rather than local defense activation in plants attacked by herbivorous insects.

### 5.4. Two Pore Channel 1 (TPC1)

Two pore channels (TPCs) are organellar cation channels that are widely expressed in animals and plants. In animals, they are localized in the endolysosomal membrane, while in plants they reside in the tonoplast of plant vacuoles [151,152,153,154]. They are members of the voltage-gated ion channel superfamily. The vacuolar TPC1 channel, also known as the slowly activating vacuole (SV) channel, has been implicated in a variety of processes in plants, including nutrient sensing, pH homeostasis, and modulation of the membrane potential. The first plant TPC1 gene was cloned in *A. thaliana* (AtTPC1), with 733 amino acids identical to the rat TPC1 sequence [152].

**Figure 6:**
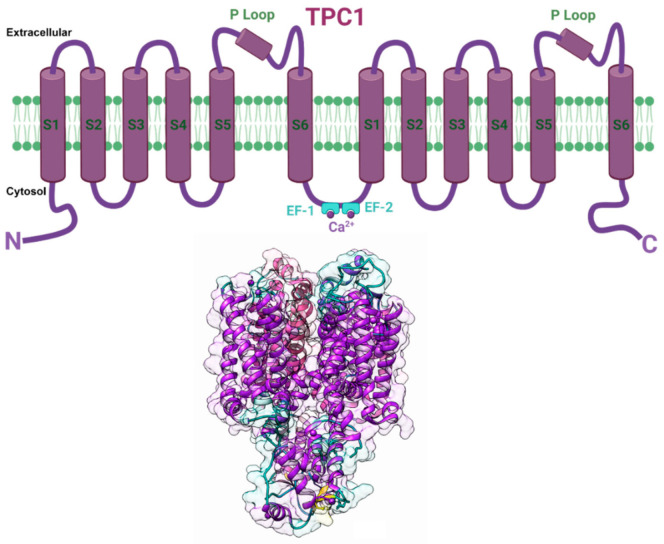
Putative structure of TPC1 channel. (Top) Schematic cartoon representation of individual plant TPC1 channel subunit comprising two repeated domains showing six membrane-spanning regions (S1–S6), two pore loops (P), and joined via a cytosolic linker containing two Ca^2+^ binding EF-hands (EF1 and EF2). (Bottom) TPC1 secondary 3D structure model showing two subunits in transparent surface view was developed from PDB structure 5DQQ (*Arabidopsis thaliana* TPC1). The image was prepared using Chimera software [122]. Created with BioRender.com (accessed on 30 August 2021).

Plant and animal TPCs are similar in sequence to voltage-gated Ca^2+^ and Na^+^ channels and feature two shaker-like units with six transmembrane domains (S1–S6), each joined by a cytosolic linker containing two Ca^2+^-binding EF-hands (EF1 and EF2). (Figure 6). Voltage and an increase in the cytosolic Ca^2+^ level both influence the activity of plant TPCs. Ca^2+^ binding to the cytosolic EF-hand domain induces conformational changes in the pair of pore-lining inner helices from the first 6-TM domains, whereas membrane potential activates the second voltage-sensing domain, which undergoes conformational changes and facilitates pore opening [155]. The SV channel transports Ca^2+^ in addition to Na^+^ and K^+^ and has a permeability ratio of 3:1 for Ca^2+^ to K^+^ [156,157]. Ca^2+^ release is substantially dependent on the concentration of cytosolic free Ca^2+^, indicating that this channel is involved in Ca^2+^-induced Ca^2+^ release [156,158]. The plant TPC1 has been implicated in insect-plant interactions. A study by Kiep et al. [98] has shown that an increase in local cytosolic Ca^2+^ and systemic Ca^2+^ response was induced in response to *S. littoralis* feeding on *A. thaliana*. By using real-time imaging in *A. thaliana* expressing the Ca^2+^ reporter aequorin to monitor the induction of local and systemic cytosolic Ca^2+^ signals, this study showed that simulated herbivory by wounding inhibited the systemic Ca^2+^ signal in the *tpc1* knockout mutant. These results indicated that the TPC1 channel plays a key role in the systemic [Ca^2+^] cyt signal induced by insect herbivory in *A. thaliana*. Another study by Vincent et al. [84] employed *A. thaliana* plants expressing the GFP-based Ca^2+^ indicator GCaMP3 to visualize Ca^2+^ accumulation in response to aphid *M. persicae* feeding. Within 95 s of the aphids settling, a robust fluorescence burst was seen, indicating cytosolic Ca^2+^ elvation. The rise in cytosolic Ca^2+^ was strongly dependent on Brassinosteroid Insensitive Associated Kinase I (BAK1), the plasma membrane Ca^2+^ permeable ion channels glutamate receptor-like 3.3 and 3.6 (GLR3.3 and GLR3.6), which are critical regulators of extracellular Ca^2+^ import into the cytoplasm of plant cells. In addition, this study also revealed that the increase in cytosolic Ca^2+^ induced TPC1 mediated vacuolar Ca^2+^ release in response to aphid feeding, suggesting that the TPC1 channel operates in conjunction with the plasma membrane Ca^2+^ permeable ion channels GLR3.3 and GLR3.6 in mediating cytosolic Ca^2+^ increase during insect herbivory [84].

### 5.5. H^+^-ATPase

The proton-pumping ATPases (H^+^-ATPases) are the primary pumps responsible for the generation of a proton gradient across cellular membranes. This electrogenic transporter uses energy from ATP hydrolysis to drive the translocation of protons against their concentration gradient from the cytosol to the external aqueous environment [159]. The H^+^-ATPase is located in the plasma membrane (PM) of plant cells. It has been demonstrated that the activation and suppression of the H^+^-ATPase activity in the plant plasma membrane modulate V_m_, resulting in the alteration of PM ion channels and transporters functions [160]. The PM H^+^-ATPase is a single 100 kDa polypeptide and a member of the large family of phosphorylation (P)-type ATPases. It is composed of six transmembrane helices (M1–M6) and a cytoplasmic domain containing phosphorylation (P), nucleotide-binding (N), and actuator (A) domains involved in ATP hydrolysis. The PM H^+^-ATPase has been implicated in various physiological processes, including cell development, intracellular pH regulation, food uptake, stomatal opening, salt tolerance, and cellular expansion [161,162,163,164,165].

Plant PM H^+^-ATPase has been shown to contribute in the propagation of the intracellular defense signaling cascade by modifying V_m_ in response to herbivore feeding [166]. A study by Camoni et al. [167] demonstrated that *S. littoralis* oral secretions effectively inhibited *Phaseolus lunatus* PM H^+^-ATPase, resulting in decreased H^+^ extrusion from the cytosol and modification of the V_m_. This observation implied that H^+^ extrusion by the plant H^+^-ATPase was involved in V_m_ regulation and might initiate a plant defensive response to herbivory. Another recent study by Kumari et al. [168] has revealed that *Arabidopsis* H^+^-ATPase 1 (AHA1) is involved in the formation of slow wave potentials (SWPs), which are required for long-distance electrical transmission during herbivore-induced plant defense. Fusicoccin, a PM H^+^-ATPase activator, prolonged the SWP repolarization phase in leaves distal to wounds. The repolarization phase was significantly prolonged in reduced function *aha1* mutants, whereas the duration of SWP repolarization was reduced in the presence of a gain-of-function mutant *ost2-2D*. Additionally, *S littoralis* larvae performed better on *aha1-7* mutants than on wild-type plants. Overall, these observations suggest that the PM H^+^-ATPase is required for the regulation of the V_m_ and electrical signal propagation between different parts of a plant during insect herbivory.

## 6. Reactive Oxygen Species (ROS)

Reactive oxygen species (ROS) are highly reactive molecules generated by redox reactions. They are part of several biological processes, such as photorespiration, oxidative phosphorylation, the electron transport chain (ETC), as well as a plant defense against pathogens and herbivores. ROS is produced in the mitochondria, chloroplast, and peroxisomes. There are several forms of ROS like superoxide anion (O^2−•−^), hydrogen peroxide (H_2_O_2_^•^), hydroxyl radical (HO^•^), peroxynitrite (ONOO), and singlet oxygen (^1^O_2_) [169]. ROS is typically produced by the nicotinamide adenine dinucleotide phosphate (NADPH) oxidase complex, which catalyzes the reduction of molecular oxygen to superoxide anion, which is then converted to H_2_O_2_. In plants, respiratory burst oxidase homologs (RBOHs) were found to be the key enzymes that catalyze the formation of ROS, which is a key step in plant protection against herbivores [170,171,172]. The respiratory burst oxidase homolog D (RBOHD) has been found to be essential for the propagation of ROS waves [173]. The significance of RBOHs in organizing responses against chewing insect herbivores was verified in *N. attenuate* where tobacco hornworm (*Manduca sexta*) OS enhanced NaRBOHD (*N. attenuata* NADPH oxidase homolog) on damaged leaves. ROS accumulation was diminished in *M. sexta* OS treated NaRBOHD-silenced *N. attenuata* plants without affecting OS-induced gene expression of defense-related genes [174].

The production of ROS is an inevitable by-product of metabolism in many cell types. Previously, it was assumed that ROS are toxic molecules that cause cellular damage to macromolecules [175]. Still, the role of ROS in plant defense has only recently emerged. It is well established that ROS can act as early defense signaling molecules that promote plant defense responses against a variety of pathogens and herbivores [54,176]. ROS act as secondary messengers that can penetrate up to 8.4 cm/min in *A. thaliana* [177]. Plants use ROS to alert the non-injured tissue about a plant attack by either releasing small quantities, which activates certain defense responses or prevent cell death by limiting the production of ROS [178]. ROS production has also been suggested to be involved in plant-microbe interactions as ROS can activate or repress the expression of defense-related genes [179,180]. The role of ROS in plant resistance to herbivores has been demonstrated in resistant and near-isogenic susceptible wheat after the attack of Russian wheat aphid (*Diuraphis noxia*). A strong burst of H_2_O_2_, as well as NADPH oxidase activity, was observed in resistant plants 3 h after infestation in comparison to susceptible plants. Treatments of plants with diphenyleneiodonium (DPI), an inhibitor of NADPH oxidase, suppressed the H_2_O_2_ production. Elevation in H_2_O_2_ levels (47%) was observed by treating resistant wheat plants with a mixture of glucose and glucose oxidase [181], suggesting that H_2_O_2_ plays a role in the defense response against *D. noxia* infestation.

Studies have shown that ROS serve as early defense signaling molecules in response to herbivore-induced wounding and secretions such as OS and oviposition. Imbiscuso et al. [182] investigated the effect of brake fern (*Pteris* *vittata*) response to herbivory by *S. littoralis*. The *P. vittata* plants responded to the attack of *S. littoralis* by activating peroxidases which produced H_2_O_2_. The concentration of H_2_O_2_ in leaves was lower in mechanically wounded young leaves than herbivory wounded leaves, suggesting that *P. vittata* can distinguish between mechanical and herbivory wounding by modulating the amount of ROS production. A study by Shinya et al. [183] demonstrated that the application of OS isolated from generalist herbivore, nightfeeding rice armyworm, (*Mythimna loreyi*)*,* caused a strong intracellular ROS generation on rice cells, and a similar effect was obtained upon application of synthetically prepared N-linolenoyl-L-Glu, the most abundant FAC present in OS of *M. loreyi,* indicating that FAC from *M. loreyi* OS promoted ROS production in rice cells.

Recently, our group Gandhi et al. [184] demonstrated that *M. sexta* oral secretions (OS) induced ROS generation in isolated tomato protoplasts. Interestingly, our study showed that the application of tomato plant-fed (PF) *M. sexta* OS enhanced ROS generation while artificial diet-fed (DF) OS could not induce ROS in tomato protoplasts, suggesting that the oral secretions of *M. sexta* play an indispensable role in inducing ROS generation in tomato protoplasts. Our study also showed that the *M. sexta* PF-OS induced ROS increase was diminished in the presence of a Ca^2+^ chelator, BAPTA-AM, suggesting that there is a link between Ca^2+^ and ROS signaling. Several lines of evidence have indicated the existence of a positive feedback mechanism between ROS and Ca^2+^ production. In a heterologous expression system, treatment with ionomycin, an ionophore that leads to Ca^2+^ influx into cells, resulting in activation of RHD2 NADPH oxidase (root hair defective 2 reduced nicotinamide adenine dinucleotide phosphate) in root tips of *A. thaliana* confirming Ca^2+^ triggered RHD2 NADPH oxidase activity. These observations suggest that Ca^2+^ acts upstream of ROS production [185].

Compelling evidence indicates that ROS production by RBOHD is dependent on the Ca^2+^ binding [186,187]. RBOHD carries 2 EF-hands which are known to participate in Ca^2+^ dependent modulation [188]. Abscisic acid (ABA) signaling in guard cells involves both Ca^2+^ and ROS. *A. thaliana* mutants lacking certain NADPH oxidases (AtRBOHD and AtRBOHF) do not close their stomata and produce ROS, Ca^2+^, and Ca^2+^ channel activation when they are exposed to ABA. Supplementation of H_2_O_2_ to guard cells rescues the mutant phenotype, implying that Ca^2+^ entry proceeds downstream of ROS generation in ABA signaling [189,190].

In *A. thaliana*, the production of H_2_O_2_ was observed in leaves 72 h after oviposition by cabbage moth (*Pieris brassicae*) and was recognized by the formation of a reddish-brown precipitate. This result indicates that oviposition can trigger a localized response that resembles the hypersensitive response induced by pathogens [191]. A recent study by Stahl et al. [192] showed that eggs of *P. brassicae* induced generation of H_2_O_2_, salicylic acid and defense gene expression in *A. thaliana*. This study also revealed phosphatidylcholines (PCs) released from eggs is the key signaling molecule that activates gene expression and triggers various defenses in the plants.

### Tools Used to Monitor ROS Signaling in Plant-Herbivore Interactions

While ROS relevance in plant-herbivore interaction is gaining momentum, the detection and characterization of ROS are still a significant bottleneck in this field. The early detection and quantification of ROS can be carried out by either utilizing genetically encoded fluorescent ROS sensors such as redox-sensitive green fluorescence protein (Ro-GFP), or synthetic fluorescent probes, such as 3,3′-diaminobenzidine (DAB) and 2′,7′-dichlorofluorescein diacetate (H_2_DCFDA). Genetically encoded ROS sensors “Ro-GFP” can monitor the cellular redox status at a high spatiotemporal resolution [193,194,195,196,197,198,199]. A recent study by Hipsch et al. [200] measured the whole plant ROS generation in response to high light, cold, and drought by using a chloroplast-targeted redox-sensitive green fluorescence protein 2 (RoGFP2). This finding suggests that whole-plant redox imaging using genetically encoded ROS sensors can be applied in a wide range of abiotic and biotic stress conditions, including plant-herbivore interaction. Despite the promising findings, the application of genetically encoded ROS sensors in plant-herbivore interactions is still limited due to the laborious and time-consuming method of its application. In contrast, synthetic fluorescent probes such as DAB and H_2_DCFDA are easier to use and can measure ROS in real-time with high sensitivity [201]. DAB has been used in many studies on plants as a reliable biomarker for reactive oxygen species (ROS) production [202,203,204]. However, in recent years, H_2_DCFDA has gained attention for its potential to measure the ROS levels in real-time on whole plants and as well as plant protoplasts [184,205,206]. Fichman et al. [205] measured the effect of light stress, injury, and pathogen, *P. syringae* pv. tomato DC 3000 on ROS signaling in H_2_DCFDA dye sprayed *A. thaliana* by using whole plant-live imaging. This study suggests that the combination of live-cell imaging and the use of H_2_DCFDA enables real-time monitoring of ROS in plants in response to various stress and pathogen treatments. This study also utilized an RBOHD (*rbohD*) knockout, and upon treatment with different stimuli, less ROS generation was observed. In contrast, another cytosolic ascorbate peroxidase 1 (*apx*) knockout produced more local as well as systemic ROS upon wounding or light stress treatments implying that this mutant had less ROS quenching capacity.

## 7. Conclusions

Recent years have witnessed immense progress in identifying the early defense signaling components in plant defense against herbivores, but studies on the molecular identification and characterization of these components are still a work in progress. However, with the advent of state-of-the-art imaging techniques, physiological and biochemical assays, and genomics may help us to understand the early defense signaling events by coordinating the plasma membrane potential changes, ion channels modulation, intracellular Ca^2+^ and ROS generation, gene expression, and, ultimately, the host plant defense response against herbivores. Transforming plants with biosensors such as GCaMP-Ca^2+^ and Ro-GFP-ROS sensors can help in the early identification of the plant defense responses. HAEs such as OS, frass, and oviposition could be used to develop strategies for early detection of the impending herbivory. So far, only a handful of Ca^2+^ permeable channels have been identified that plays a role in plant-herbivore interactions. Further studies are needed to unravel other ion channels that may be contributing to the modulation of V_m_, Ca^2+^, and ROS, the downstream signaling cascade, and, more importantly, the role of these ion channels in triggering a rapid defense response. A deeper understanding of these early signaling events will eventually help us to minimize herbivory by developing pest management strategies based on plant-herbivore monitoring systems. Such knowledge can be instrumental in the design of plants with improved resistance against herbivores. As such, in the future, it will be important to develop effective small-molecule modulators that can inhibit or enhance the early signaling events in plant-herbivore interactions. Such an approach would not only facilitate research on early plant signaling events but also help in developing novel strategies for the development of herbivore-resistant crops.

## Data Availability

Not applicable.
